# Combining EEG and MEG for the Reconstruction of Epileptic Activity Using a Calibrated Realistic Volume Conductor Model

**DOI:** 10.1371/journal.pone.0093154

**Published:** 2014-03-26

**Authors:** Ümit Aydin, Johannes Vorwerk, Philipp Küpper, Marcel Heers, Harald Kugel, Andreas Galka, Laith Hamid, Jörg Wellmer, Christoph Kellinghaus, Stefan Rampp, Carsten Hermann Wolters

**Affiliations:** 1 Institute for Biomagnetism and Biosignalanalysis, Westfälische Wilhelms-Universität Münster, Münster, Germany; 2 Department of Neurology, Klinikum Osnabrück, Osnabrück, Germany; 3 Ruhr-Epileptology Department of Neurology, Universitätsklinikum Knappschaftskrankenhaus Bochum, Bochum, Germany; 4 Department of Clinical Radiology, Universitätsklinikum Münster, Münster, Germany; 5 Department of Neuropediatrics, Universitätsklinikum Schleswig-Holstein, Kiel, Germany; 6 Epilepsy Center, Department of Neurology, Universitätsklinikum Erlangen, Erlangen, Germany; University College of London - Institute of Neurology, United Kingdom

## Abstract

To increase the reliability for the non-invasive determination of the irritative zone in presurgical epilepsy diagnosis, we introduce here a new experimental and methodological source analysis pipeline that combines the complementary information in EEG and MEG, and apply it to data from a patient, suffering from refractory focal epilepsy. Skull conductivity parameters in a six compartment finite element head model with brain anisotropy, constructed from individual MRI data, are estimated in a calibration procedure using somatosensory evoked potential (SEP) and field (SEF) data. These data are measured in a single run before acquisition of further runs of spontaneous epileptic activity. Our results show that even for single interictal spikes, volume conduction effects dominate over noise and need to be taken into account for accurate source analysis. While cerebrospinal fluid and brain anisotropy influence both modalities, only EEG is sensitive to skull conductivity and conductivity calibration significantly reduces the difference in especially depth localization of both modalities, emphasizing its importance for combining EEG and MEG source analysis. On the other hand, localization differences which are due to the distinct sensitivity profiles of EEG and MEG persist. In case of a moderate error in skull conductivity, combined source analysis results can still profit from the different sensitivity profiles of EEG and MEG to accurately determine location, orientation and strength of the underlying sources. On the other side, significant errors in skull modeling are reflected in EEG reconstruction errors and could reduce the goodness of fit to combined datasets. For combined EEG and MEG source analysis, we therefore recommend calibrating skull conductivity using additionally acquired SEP/SEF data.

## Introduction

Epilepsy surgery is an important option to treat pharmaco-resistant focal epilepsy and its success depends heavily on the correct determination of the epileptogenic zone. The epileptogenic zone is defined as “the minimum amount of cortex that must be resected (inactivated or completely disconnected) to produce seizure freedom” [1]. It is estimated prior to surgery by considering information available from initial seizure semiology, lesions in magnetic resonance images (MRI), video and electroencephalography (EEG) long-term monitoring, magnetoencephalography (MEG), single photon emission computed tomography (SPECT), positron emission tomography (PET), neuropsychological examination and others. The *irritative zon*e, one of the important zones for locating the epileptogenic zone, is identified by EEG and/or MEG. The irritative zone is defined as the brain area producing synchronous discharges of nerve cell clusters between seizures (interictal). The identification of the irritative zone has not only localizatory, but also prognostic value [2]–[4]. Multifocal or contralateral epileptic discharges are correlated to a less favorable postoperative outcome regarding seizure freedom [3]. Although the irritative zone might not always be identical to the epileptogenic zone, as in some patients with bitemporal spikes that became seizure free after the resection of one temporal lobe [1], it holds important information regarding the location of the epileptogenic zone. An accurate identification of the irritative zone can therefore be of high importance. We propose here a new experimental and methodological source analysis pipeline for the non-invasive identification of this zone using combined or single modality EEG/MEG source analysis in a calibrated realistic head model. The methodology will be applied in a case study with a patient suffering from refractory focal epilepsy who showed a sufficient amount of interictal spikes for the purpose of this study. In this way, we will be able to show the advantages, but also the risks of combined EEG/MEG and single modality EEG or MEG source reconstructions of interictal epileptic activity and will point out a guideline how to minimize the risks when working with simultaneously acquired data. Our study thus contributes to important and long-standing questions on feasibility and accuracy of combined EEG/MEG versus single modality EEG or MEG source reconstruction not only with regard to applications in epileptology, but also more generally for neuroscientific studies.

Since some decades, efforts have been made to reconstruct the electrical activity in the human brain that is underlying the measured EEG and/or MEG. The reconstruction of the so-called primary currents is called the inverse problem of EEG/MEG. As a model for the primary current, most of the studies use the mathematical current dipole model, although multipoles have also been studied [5]. The solution to the inverse problem requires repeated simulations of the field distribution at the head surface for a given current source in the brain, the EEG/MEG forward problem. While existence and uniqueness of the solution for dipole sources have been proven for the forward problem [6], the inverse problem is non-unique [7] leading to a variety of inverse reconstruction algorithms that are based on different a-priori assumptions. Inverse reconstruction algorithms are sensitive to deficiencies in lead field accuracy, i.e., deficiencies in head volume conductor modeling within the EEG/MEG forward problem. Non-invasive source analysis has already emerged as a promising tool in presurgical epilepsy diagnosis [8]–[17]. The results of EEG source analysis were shown to avoid or guide intracranial EEG recordings, and proved to be a key element in the surgical decision process in a significant percentage of patients [9]. Moreover, epileptogenic temporal subregions could be identified using EEG source reconstruction [18]. In frontal lobe epilepsy, it was reported that MEG was more successful for screening and localizing than EEG [19]. MEG was furthermore shown to help characterizing potentially epileptogenic lesions and pointing intrinsic epileptogenicity of malformations of cortical development [20]–[22]. While most source analysis studies used either EEG or MEG data, we will present here a new strategy for combined EEG/MEG source reconstruction and apply it for the first time to the data of an epilepsy patient.

EEG and MEG contain complementary information. With regard to the detection of epileptic discharges, [23]–[25] reported that some spikes could be recorded only with MEG and not with EEG and vice versa. In [26] the mathematical notation of this complementarity was given and it was shown that for a continuously distributed neuronal current, information missing in EEG is precisely the information that is available in MEG, and vice versa. Because of this complementarity, the combined analysis of EEG and MEG data is of increasing interest and might lead to more stable source reconstructions and a superior spatial resolution [27]–[32]. It is furthermore motivated by the fact that MEG can almost only measure quasi-tangentially oriented sources, while EEG is more sensitive to the quasi-radial neural generators, and under the constraint of an appropriate volume conductor model, reveals a better depth resolution [7], [27]–[29], [31], [33]. For a tangential dipole source and a sufficient signal to noise ratio (SNR), MEG field topography generally is very similar to EEG potential topography rotated by 90 degrees with a smaller distance between the poles due to the blurring effect of the skull compartment on EEG. This rotation increases the probability that at least one modality will measure both poles of a dipolar field pattern, an essential prerequisite for a successful source analysis, thus reducing difficulties caused by limited sensor coverage. Various studies have shown that EEG is especially sensitive to geometry and conductivity of the skull, while the MEG is nearly not affected by inaccurate modeling parameters for this compartment [7], [33]. Recent studies using EEG source analysis suggest distinguishing compact and spongy bone tissues in order to account for the local variations (inhomogeneity) of the skull [34]–[37] and modeling skull holes [38]. Both EEG and MEG are sensitive to errors in the representation of the tissue properties of all compartments which are bounded by the inner skull surface, e.g., the highly conducting cerebrospinal fluid (CSF) [33], [39], [40] and the anisotropic brain tissues [32], [41], [42]. A combined EEG/MEG source analysis should therefore consider the different sensitivity profiles. In this study, we propose calibrated realistic six compartment (skin, skull compacta, skull spongiosa, CSF, gray and white matter) anisotropic (for the brain) head modeling using the finite element method (FEM). The FEM allows high flexibility in modeling the EEG and MEG forward problem in geometrically complicated inhomogeneous and anisotropic head volume conductors (see recent review in [6]). In this way, we expect to significantly improve the synergistic effects of EEG and MEG, leading to more reliable source reconstructions not only in the field of presurgical epilepsy diagnosis but also in other application fields of source analysis.

This is the first source analysis study for simultaneously measured EEG and MEG of epileptic activity using an individual, conductivity calibrated six compartment high resolution FE model of the patient's head. The conductivity of the skull is estimated using a calibration method based on somatosensory evoked potential (SEP) and field (SEF) measurements, one additional run that preceded the acquisition of multiple runs of epileptic discharges. Our study design allows us to investigate the influence of the number of compartments (six versus the standard three compartment approach) as well as that of compartment conductivities (individually calibrated versus standard skull conductivity parameters as found in the literature) on the localization of somatosensory evoked responses and interictal epileptic activity.

## Patient and Methods

### 1.1 Ethics Statement

The patient and her parent signed written consent forms and all procedures have been approved by the ethics committee of the University of Erlangen, Faculty of Medicine on 10.05.2011 (Ref. No. 4453).

### 1.2 The patient

The patient in this study is a 17 year old female suffering from pharmaco-resistant focal epilepsy since 11 years. Apart from her sister suffering also from focal epilepsy, she does not have any further risk factors. Several 3 Tesla MR acquisitions, following protocols sensitive to epileptogenic lesions, were negative. An FDG-PET scan showed a diffuse and extended left fronto-temporal hypometabolism. Interictal discharges have been recorded in EEG and MEG, most of them over the left temporal regions and only few over the left frontal region.

### 1.3 MRI measurements

T1-weighted (T1w-), T2-weighted (T2w-) and diffusion-tensor (DT-) MRI scans were acquired with a 3T scanner (Gyroscan Intera/Achieva 3.0T, System Release 2.5 (Philips, Best, NL)). A 3D-T1w gradient-echo pulse sequence with inversion prepulses, TR/TE/TI/FA = 9.2 ms/4.4 ms/1014 ms/9°, with water selective excitation and cubic voxels with 1.17 mm edge length, and a 3D-T2w TSE pulse sequence, TR/TE = 2000 ms/378 ms, cubic voxels, 1.17 mm edge length, were used. MR images were resampled to 1 mm isotropic resolution, used as the resolution of the FE mesh throughout this study. DT-MRIs (DTI) were acquired using a Stejskal-Tanner spin-echo EPI sequence, TR/TE = 7546 ms/67 ms. Geometry parameters were: FOV 240×240 mm for 70 transverse slices, 1.875 mm thick without gap, square matrix of 128, i.e. cubic voxels with 1.875 mm edge length. One volume was acquired with diffusion sensitivity b = 0 s/mm^2^ (i.e. flat diffusion gradient) and 20 volumes with b = 1000 s/mm^2^ for diffusion weighting gradients in 20 directions, equally distributed on a sphere. Geometry distortion due to susceptibility gradients was maximal in phase encoding direction (anterior-posterior), bandwidth 20.3 Hz/pixel. An additional data set with only flat diffusion gradients and reversed spatial encoding gradients was acquired for distortion correction according to [43]. The total amount of acquisition time required for T1w-, T2w- and DT-MRI scans was 27 minutes (approximately 9 minutes each).

### 1.4 Head model generation

#### 1.4.1 Registration and segmentation of T1w and T2w MRI

**Figure 1:**
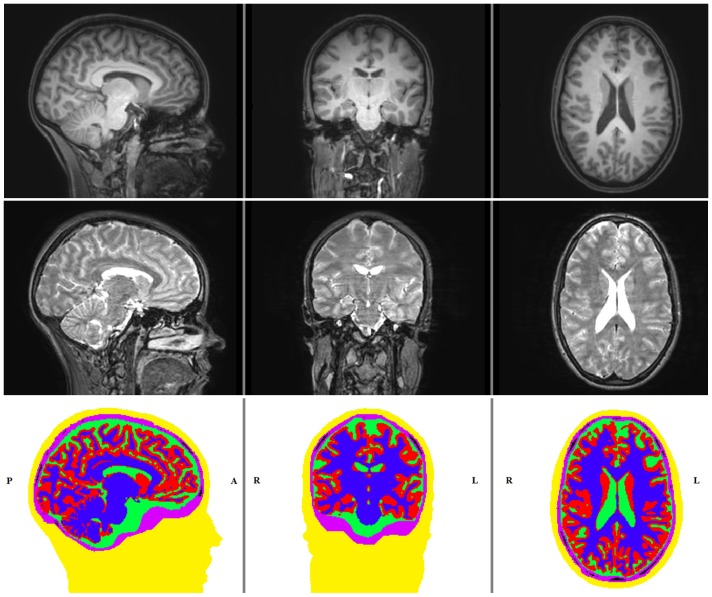
T1-w, T2-w MRI and the segmented image. Sagittal (left column), coronal (middle column) and axial (right column) slices of T1w-MRI (top row), T2w-MRI (middle row) and the 6 compartment segmentation result showing the head tissues skin (yellow), skull compacta (purple), skull spongiosa (black), CSF (green), gray matter (red) and white matter (blue) (bottom row).

In a first step, the T1w-MRI was resampled to obtain an isotropic resolution of 1 mm. The T2w-MRI was registered onto the T1w-MRI using a rigid registration approach and mutual information as a cost-function as implemented in FSL (http://www.fmrib.ox.ac.uk/fsl). Then, brain, inner skull, outer skull and extracranial tissue (summarized in the following as the *skin* compartment) masks were obtained from the T1w and T2w images following [44]. In a next step, the T1w image served for the segmentation of gray and white matter and the T2w image for the segmentation of the CSF using a hidden Markov random field model [45]. All these steps were realized using FSL software. The gray matter mask was then further improved using Freesurfer (http://surfer.nmr.mgh.harvard.edu). The skull segmentation was visually inspected and manually corrected using CURRY (http://www.neuroscan.com/curry.cfm). The skull spongiosa was segmented from the T2w-MRI using a threshold based segmentation restricted within the skull compartment. The resulting segmentation is shown in Figure 1.

#### 1.4.2 Generation of the geometry-adapted hexahedral finite element mesh

A hexahedral finite element mesh was constructed out of the labeled volume. In order to increase conformance to the real geometry and mitigate the staircase effects of a voxel mesh, we shifted the nodes on material interfaces [46]. This approach was validated for EEG source analysis in multi-layer sphere models, leading to significant error reductions compared to regular hexahedral approaches [47] and high numerical accuracies especially for high-resolution meshes [48]. We chose a node-shift factor of 0.33 to ensure that interior angles at element vertices are convex and the Jacobian determinant in the FEM computations remains positive. This procedure resulted in a geometry-adapted hexahedral FE mesh with 3,993,881 vertices and 3,895,971 elements. The software SimBio-VGRID (http://www.rheinahrcampus.de~medsim/vgrid) was used for mesh generation.

#### 1.4.3 Inclusion of gray and white matter conductivity tensors

The DTI was corrected for eddy current (EC) artifacts by affinely registering directional images to the image with flat diffusion gradients using the FSL routine FLIRT. Subsequently, the gradient directions were reoriented using the rotational part of the transformation matrices obtained during the EC correction scheme. Then we applied our diffeomorphic approach for nonlinear correction of susceptibility artifacts in the DTI dataset according to [43]. This approach has been implemented in the freely-available SPM (http://www.diffusiontools.com/documentation/hysco.html) and FAIR (http://www.mic.uni-luebeck.de/people/jan-modersitzki/software/fair.html) software packages. The nonlinearly corrected DT images were later rigidly registered to the T2w image and corresponding gradient directions were reoriented accordingly [43]. Following this, diffusion tensors were calculated using the FSL routine DTIFIT [49].

**Figure 2:**
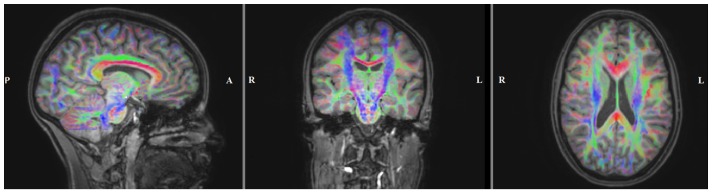
Diffusion directions obtained from DTI. Sagittal (left), coronal (middle) and axial (right) slice of the color coded fractional anisotropy (FA) map computed from the registered diffusion tensors and plotted on the registered T1w-MRI. The color indicates the main fiber orientation: red is left-right, green is anterior-posterior and blue is superior-inferior.

Figure 2 shows the result of the DTI preprocessing and registration procedure overlaid on the T1w-MRI. As a last step, conductivity tensors were calculated from the artifact-corrected and registered DTIs using the effective medium approach as described in [50], [51] and embedded into the geometry-adapted hexahedral FE head model.

#### 1.4.4 Head models

We used a variety of head models and their properties are illustrated in Table 1.

**Table 1:** Head models used in this study: The first column indicates the name of the head model and the remaining columns the conductivities for the respective tissue compartments.

Head Model	Skin	Skull Compacta	Skull Spongiosa	Skull	CSF	Gray Matter	White Matter	Brain
*6C_Cal*	0.43[^51^]	0.0024	0.0084	/	1.79 [^39^]	#	#	/
*6C_41*	0.43	0.0041[^29^], [^53^]	0.0147	/	1.79	#	#	/
*6C_70*	0.43	0.0070[^34^]	0.0251[^34^]	/	1.79	#	#	/
*6C_132*	0.43	0.0132[^54^]	0.0471	/	1.79	#	#	/
*6C_330*	0.43	0.0330[^55^]	0.1179	/	1.79	#	#	/
*3C_Cal*	0.43	-	-	0.0024	-	-	-	0.33[^29^], [^53^]
*3C_100*	0.43	-	-	0.0100[^35^]	-	-	-	0.33

The compartments that were (were not) distinguished are indicated by a slash (dash) sign. Isotropic conductivity values were taken from the literature [29], [34], [35], [39], [52]–[55] or were determined in our SEP/SEF skull conductivity calibration procedure (*_Cal models; see sections 1.11 and 2.1). # indicates that conductivity tensors were determined as described in section 1.4.3. In all 6C models, the ratio of skull spongiosa to compacta was kept constant to the mean of the ratio measured by [34].

Our most realistic head models contain the six compartments (models *6C* in Table 1) skin, skull compacta, skull spongiosa, CSF, and brain gray and white matter. We use conductivity values of 0.43 S/m for the compartment skin [52] and 1.79 S/m, which was average over 7 subjects, ranging in age from 4.5 months to 70 years, with a standard deviation of less than 1.4% between subjects and for frequencies between 10 and 10,000 Hz, for the highly conductive CSF [39]. Conductivity modeling of gray and white matter has been described in section 1.4.3. An accurate modeling of the human skull is essential for the success of simultaneous EEG/MEG source analysis [29], [30]. However, it is discussed quite controversially in the literature, motivating the inclusion of various models in our investigations: Model *6C_70* (suffix indicates the compacta conductivity in S/m*10^−4^) uses the average of conductivities measured for skull compacta and spongiosa over four patients [34]. All of our 6C models use a fixed ratio of spongiosa∶compacta of about 3.6∶1 (mean of the measurements of [34]). The value of 0.0041 S/m was used in [53] and is implemented as a standard skull conductivity in most commercial source analysis packages (model 6C_41 in Table 1) [29] (to be precise, [29] used 0.0042 S/m). Model 6C_132 is motivated by simultaneous intra- and extra-cranial potential measurements from five epilepsy patients [54] and model 6C_330 by [55].

The standard low-parametric three compartment (*3C*) isotropic volume conductor model (skin, skull, brain) is still frequently used in source analysis (see recent review in [6]). It is, therefore, instructive to compare 6C and 3C results. For the homogenized brain compartment in the 3C models, we chose a conductivity value of 0.33 S/m [29], [53]. The skull conductivity value of 0.01 S/m in model *3C_100* was found as an optimal choice to approximate the skull's layeredness in compacta and spongiosa, in a globally isotropic skull modeling approach (in [35], average over four subjects). Finally, the generation of the calibrated head models *6C_Cal* and *3C_Cal* is explained in section 2.1.

### 1.5 EEG and MEG measurements

74 channel EEG (plus additional 6 channel EOG to detect eye movements) and 275 channel whole head MEG (plus 29 reference channels to calculate synthetic gradiometers) (CTF, VSM MedTech Ltd.) along with ECG were simultaneously acquired in a magnetically shielded room. Prior to measurements, the electrode positions were digitized using a Polhemus device. In order to minimize head movement and to ensure patient comfort, EEG/MEG data were acquired in supine position. Since MRI was also measured in supine position, we thereby prevent brain shift and the resulting small changes in CSF layer thickness due to differences in subject's position between MRI and EEG/MEG measurements, which were shown to have a significantly negative effect on source reconstruction results [56]. During the measurements, head movement had been continuously tracked with three head localization coils and only the runs with maximum head movement lower than 8 mm were accepted for further analysis.

#### Somatosensory evoked potentials (SEP) and fields (SEF)

Somatosensory evoked potential and field measurements were carried out for calibrating the volume conductor model with the goal of improving EEG and especially combined EEG/MEG source analysis (see also section 1.10). For this purpose, the median nerve of the patient's left wrist was stimulated using square electrical pulses with 0.5 ms duration. The stimulus strength was adjusted to see a clear movement of the thumb. The inter-stimulus interval was varied randomly between 350 to 450 ms to avoid habituation and to obtain a clear pre-stimulus interval. A reduction in stimulus artifacts was achieved by reversing the polarity of the stimulation during the second half of the measurement. Within this 7 minutes long run 950 events were recorded. The data was acquired with 1200 Hz sampling rate and filtered online with a 300 Hz low pass filter.

#### Spontaneous measurements of interictal epileptic activity

The patient was advised to relax and close the eyes. This part of the measurements comprised five 8 minute long runs which were recorded with, 2400 Hz sampling rate and a 600 Hz real-time low pass filter.

### 1.6 Interictal spike marking and clustering

The spontaneous measurements were examined and epileptic spikes were marked by 3 clinical reviewers (PK, CK, SR). Custom Matlab (The MathWorks, Inc., Natick, Massachusetts, United States) code ensured that every hand-positioned marking was moved right to the peak of the maximum negativity of each epileptic spike, thereby, ensuring all markings to be at the same propagation phase of the epileptic activity [57]. Although automatic methods for spike clustering have been suggested in literature [58], in clinical practice the spikes are usually clustered according to the electrode where the maximum negativity occurs in the referential montage (common average). For example, a spike with maximum negativity at the F9 electrode is clustered as an F9 type epileptic spike. It is also common to use bipolar montages and a subsample of electrodes for reviewing the data. This means that the electrode that is selected as showing maximum negativity might not be actually the one with maximum negativity with regard to the whole electrode cap; it is just the one with maximum in the selected montage. This procedure might end up in wrong clustering of the spikes. Therefore, in our evaluation, all spikes marked by the evaluators were checked using custom Matlab code and they were clustered according to the electrode with maximum negativity in the referential montage (common average) over the whole electrode cap. To avoid clustering errors due to noise, only the electrodes in the neighborhood of the electrode selected by the clinician were used in the clustering algorithm. The neighbors were determined by first calculating the Euclidian distance of each electrode to the defined electrode and then by selecting the closest eight (eight because each electrode in our cap has eight neighboring electrodes, except the ones at the borders).

### 1.7 Pre-processing of EEG and MEG data

#### Somatosensory evoked potentials and fields

The measurements were filtered using a band pass filter of 20–250 Hz [59] and a notch filter for the line voltage frequency 50 Hz and its harmonics. Epochs of 100 ms before and 200 ms after stimulus were cut from the continuous data. After deselecting the bad channels, the epochs with artifacts in either modality were excluded using a threshold-based semi-automatic procedure followed by manual inspection. The remaining epochs were averaged, resulting in an SNR of 11.3 for the EEG and 14.4 for the MEG.

#### Spontaneous measurements of interictal epileptic activity

The spontaneous measurements were filtered using a band pass filter of 1–100 Hz [16] and a notch filter for the line voltage frequency 50 Hz and its harmonics. The manual spike markings obtained from the 3 clinical reviewers were peak corrected and clustered as described in section 1.6. The spikes were epoched from 200 ms before to 500 ms after the peak. The bad channels (TP9, TP10 and F2) and epochs were deselected manually.

### 1.8 Forward approach

In the literature, various approaches have been developed to model the source singularity, and thus to solve the EEG and MEG forward problem using the finite element method (FEM): the subtraction approach [6], [47], [60], [61], the partial integration direct approach [61], [62] and the Venant direct approach [47], [63]. In this study, we used the Venant approach based on a comparison of the performance of all three, which suggested that, for sufficiently regular meshes, the Venant approach yields suitable accuracy over all realistic source locations [47], [48]. This approach has the additional advantage that the resulting FEM approach has a high computational efficiency when used in combination with the FE transfer matrix approach [6]. Further speedup was achieved using an Algebraic MultiGrid preconditioned Conjugate Gradient (AMG-CG) solver [6]. We used standard piecewise trilinear basis functions and performed our computations using SimBio (https://www.mrt.uni-jena.de/simbio, the integration into Fieldtrip: http://fieldtrip.fcdonders.nl/development/simbio).

### 1.9 Inverse approach

In this study we used Single Dipole Deviation Scans (SDDS) [29] (also known as goal function scans) for inverse calculation to estimate the origins of single spikes. This allowed us to analyze the resulting spike clusters with regard to their centroids and focality as described in the next section. Our choice was based on [64], [65] which showed the activated cortical areas during sharp waves to be very focal with their spatial positions changing in a dynamic manner. The appeal of the SDDS was, thus, the spread of the localizations might give an estimate on the focality of the irritative zone as also proposed by [66], [67]. In the SDDS procedure, the residual variance (RV), i.e. the squared deviation, of the best fitting dipole to the measurement data was calculated for all source space locations ([29], equation (17)). In this study, EEG and combined EEG/MEG SDDSs were not regularized, while MEG SDDSs were regularized according to [29] to suppress the influence of spatially high frequent data noise that might otherwise be strongly amplified in high amplitudes of reconstructed radial source orientations. The goodness of fit (GOF) was then calculated as GOF = 1−RV and, in the results section, both RV and GOF values were given as percentages. The goal of the SDDS procedure was to determine the source space location with minimal RV and thus maximal GOF value.

EEG and MEG measure different quantities so that the units of the measurements are different. In order to perform a combined analysis both modalities need to be transferred to a common space. Here we used the SNR based transformation as suggested in [29]. In this method the data was whitened according to the noise level (calculated from the pretrigger interval where only spontaneous activity occurs) of each channel so that unitless measures for EEG and MEG were obtained to be used in a combined procedure.

### 1.10 Source reconstruction

A source space in the gray matter compartment with 2 mm resolution and 13,468 source space nodes was constructed. We used a custom Matlab code to ensure that all sources were located inside the gray matter compartment and sufficiently far away from white matter, CSF and bone tissue so that, for each source space node, the closest node of the FE mesh only belongs to elements, which are labeled as gray matter. We refer to this condition as *Venant condition*. It must be fulfilled to avoid unrealistic source modeling and numerical problems for the chosen Venant dipole modeling approach [48].

The interval from 200 to 70 ms before the spike peak was selected for noise estimation in order to determine the SNR. The peak of the spike was selected as zero time point. The EEG and MEG lead fields were calculated with SimBio and then imported to CURRY for source reconstruction. We then performed SDDS inverse reconstructions for EEG, MEG and combined EEG/MEG for single spikes at time-point −13 ms which corresponds to the middle of the rising flank for the averaged spikes [68].

Centroid spike positions were calculated as the locations where the sum of the (Euclidian) distances of SDDS localizations to the centroid was minimal. Spread spheres were used to visualize the extent of the spread of single spike reconstructions. The following algorithm describes our chosen procedure in more detail:

#### Algorithm 1 (Computation of spike cluster centroid and spread sphere):

1. Perform SDDS for all spikes with SNR>3 of one spike cluster in a predefined head model.
2. Select the SDDS reconstructions which satisfy GOF>91%.
3. Calculate the centroid position and the distances of each SDDS reconstruction in this cluster (that passes step 2) to the centroid. Determine the mean distance *m* and its standard deviation *std*.
4. If the distance of any SDDS reconstruction to the centroid exceeds *m*+2(*std*), exclude this one from the cluster.
5. Calculate the final centroid using the reconstructions from step 4 and the spread spheres using the centroid location as the center and (*m*+*std*) as the radius.

### 1.11 Skull conductivity calibration procedure using SEP and SEF data

As shown above, skull conductivity has been discussed quite controversially in the literature (see Table 1). However, an appropriate choice of it is crucial for successful source analysis of EEG and combined EEG/MEG data. While EEG source reconstructions are strongly influenced by changes in skull conductivity, the MEG is shown to be far less susceptible to it [7], [33]. In this section we explain a calibration procedure which benefits from the different sensitivity profiles of the EEG and MEG in order to individually determine skull conductivity using the SEP and SEF data of the patient. Results of computer simulation studies for validating the approach and the first application to somatosensory evoked responses from a healthy subject were presented in [69] for single modality EEG and in [70] for combined EEG and MEG. In our procedure we selected the peak of the mean global field power in the SEP/SEF-N20 component because of the simplicity of the underlying source structure: a superficial (thus high SNR in both modalities) single equivalent current dipole in somatosensory 3b area with mainly quasi-tangential source orientation (see [7], [29] and references therein). The 100 ms pre-trigger interval was used for noise estimation for both SEP and SEF datasets. Our calibration procedure can then be summarized by means of the following algorithm:

#### Algorithm 2 (SEP/SEF skull conductivity calibration):

1. Define a discrete set of skull conductivity parameters: **^∑^** = {σ_1_, σ_2_,…, σ_n_}
2. For each head model with skull conductivity parameter σ_i_, i = 1,…,n:
  - Perform SEF SDDS and calculate location *x*, orientation *o_1_* and magnitude *m_1_* of the underlying current dipole source.
  - Keep location *x* fixed and calculate *o_2_* and *m_2_* using a least squares fit to the SEP data.
  - Keep *x* and *o_2_* fixed, calculate *m_3_* using a least squares fit to the SEF data.
  - For the dipole with location *x*, orientation *o_2_* and magnitude *m_3_* calculate RV to the SEP data.
3. Select the conductivity that gives the lowest RV in step 2.d).

In step 2.a), our procedure uses the strength of the MEG to appropriately localize the primary somatosensory cortex even for less suitable skull conductivity parameters. Step 2.b) is necessary since *o_1_* and *m_1_* might be spurious in the case that the source is not optimally quasi-tangential. It uses the strength of the EEG to appropriately determine the source orientation. However, in case of inappropriate skull conductivity, *m_2_* will be spurious so that the SEF data are needed to determine the source magnitude in step 2.c).

## Results

The results section is divided into two subsections. In the first subsection, the skull conductivity calibration procedure based on the somatosensory evoked responses is carried out to determine individually optimized head models. The head models are then used in source analysis scenarios for the somatosensory evoked responses as well as, in subsection two, for evaluating the epileptic activity using single modality EEG or MEG or combined EEG/MEG source analysis scenarios.

### 2.1 Skull conductivity calibration and source analysis of the somatosensory evoked responses

**Figure 3:**
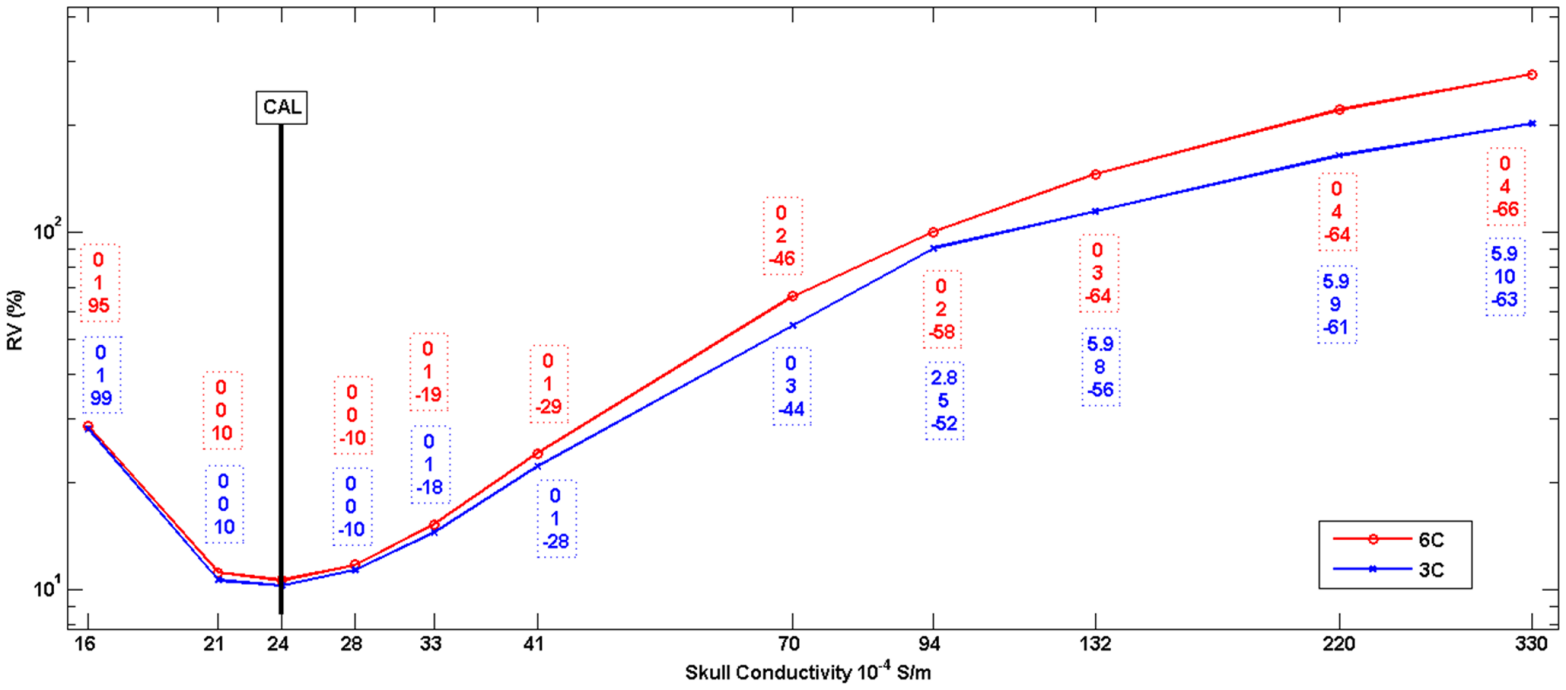
Skull conductivity calibration graph. RV (in %) obtained from Algorithm 2 in step 2.d. for different skull conductivity parameters for 6C (red) and 3C (blue) head models. The differences to the calibrated head models *6C_Cal* and *3C_Cal* (indicated by the black bar, see also Table 1) in source reconstruction are indicated by boxes with dashed frames: Difference in source location x (top row, in mm), orientation o_2_ (middle row, in degree) and strength m_2_ (bottom row, in %).

Table 1 and Figure 3 show the results of Algorithm 2 for the six compartment (head model *6C_Cal* in Table 1) and the three compartment (*3C_Cal* in Table 1) head models. In step 1 of Algorithm 2, we used a set of 11 different conductivity parameters in the range between 0.0016 S/m [71] and 0.033 S/m [55] (x-axis in Figure 3). In Figure 3, the differences in source reconstruction to the calibrated head models (indicated by the bar) when using other skull conductivity parameters are indicated by boxes with dashed frames: Differences are shown in source location *x* (top row, in mm), orientation *o_2_* (middle row, in degree) and strength *m_2_* (bottom row, in %). As expected, the source location *x* (from SEF in step 2.a)) and the orientation *o_2_* (from SEP in step 2.b)) of Algorithm 2 are hardly depending on the skull conductivity parameter, while skull conductivity, RV, and source strength *m_2_* are closely related to each other.

The value of our calibration procedure can be further appreciated by studying the sensitivity of single modality SEP or SEF source analysis to changes in volume conductor modeling. We therefore used the *6C_Cal* SDDS results as reference, and compared these to the reconstructions with other head models from Table 1. We first examined this for the *6C_70* head model. While with a source localization difference of 7.2 mm (into the depth), an orientation change of 24 degrees and a magnitude reduction by 35%, SEP source analysis depends significantly on skull conductivity, SEF reconstructions were hardly affected (differences: 0 mm, 3.7 degrees, 2%). Using head model *3C_100* led to differences of 7.2 mm, 8.9 degrees and a magnitude reduction by 60% for the SEP, and to 4.9 mm, 25.3 degrees and a magnitude reduction by 23% for the SEF. When head model *3C_Cal* was used, these differences for the SEP data fell to 0 mm, 6.9 degrees and 21% magnitude reduction, while the differences for the SEF data remained at a similar level with 4.9 mm, 25.8 degrees and 12% magnitude reduction.

### 2.2 Evaluation of interictal epileptic activity

#### 2.2.1 Interictal spike marking, clustering and SNR improvement

Our following investigations with regard to the evaluation of the epileptic activity focus on two left temporal spike types, with a maximum negativity at either FT9 or F9 electrodes, because of their high incidence. The 3 evaluators marked a total of 568 spikes and our clustering algorithm from section 1.6 determined 350 FT9 and 218 F9 spikes.

**Figure 4:**
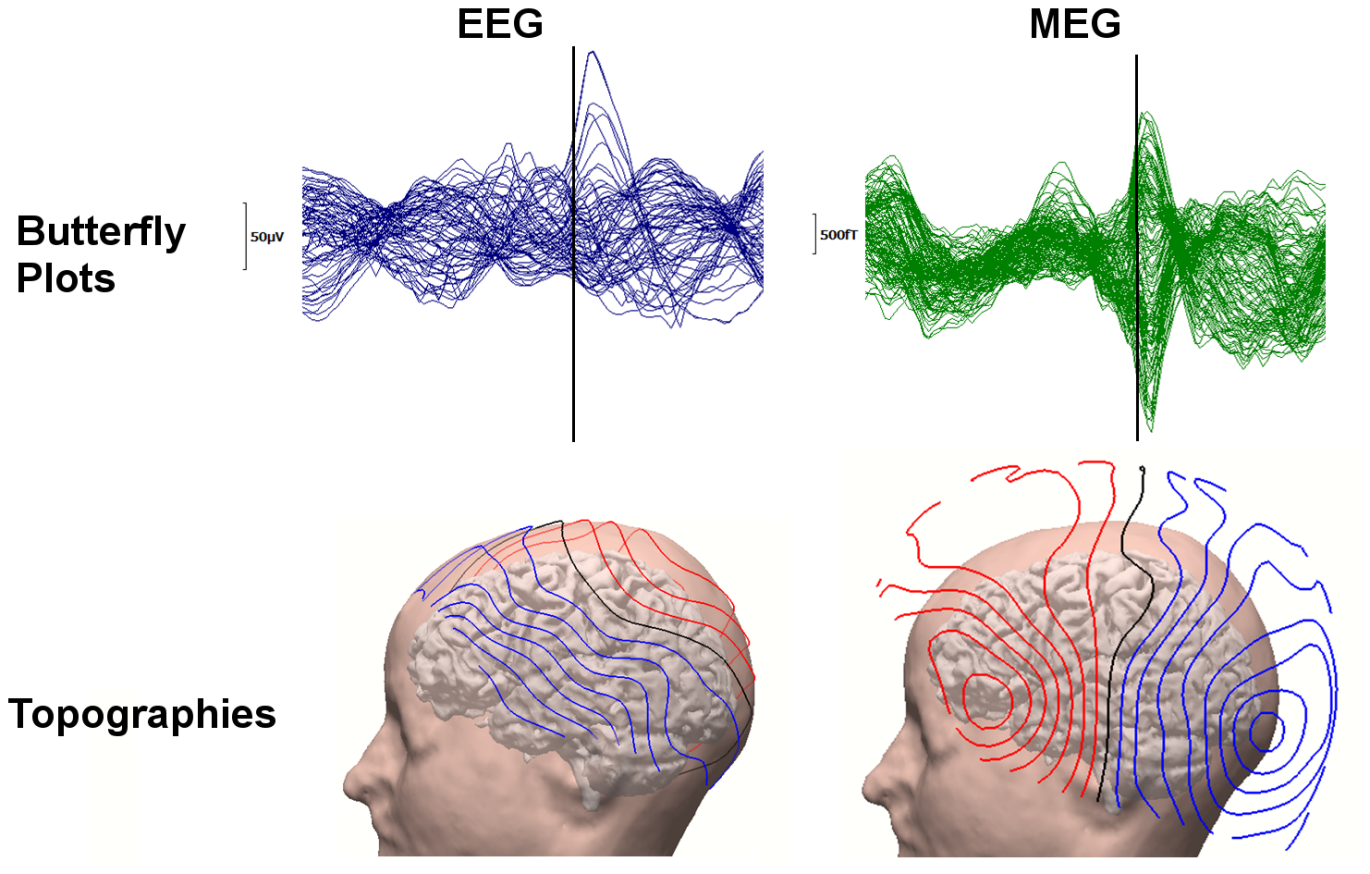
The waveform and topography of an example epileptic spike. FT9 spike: 71 channel EEG (left column) and 129 channel MEG (right column) butterfly plots (upper row, time-point −13 ms marked with a black line) and corresponding topographies from left view at time-point −13 ms plotted on individual brain and skin (bottom row).

A typical FT9 spike and its corresponding topographies for EEG and MEG can be seen in Figure 4. While we used all electrodes for EEG spike SDDS source reconstructions, for MEG only 129 gradiometers over the left hemisphere were taken into account. This subselection has been carried out to improve the SNR of the MEG spike data and the GOF of the MEG SDDSs. The SNR and GOF improvements were only possible for the MEG because the MEG spike dipolar patterns were more focal with both negative and positive poles included in the chosen subset of MEG sensors, thus reducing effectively the influence of the spontaneous activity from the right brain hemisphere, while for the EEG, the spike negative and positive poles were in different hemispheres.

#### 2.2.2 Effects of varying skull conductivity on source reconstruction for the epileptic activity

In this section the effects of varying skull conductivity on EEG or MEG source reconstruction of FT9 and F9 spikes are investigated. Therefore, we used Algorithm 1 to compute the centroids and spread spheres for these two spike clusters using the six compartment head models from Table 1. In order to focus on skull modeling effects, we employed here the GOF selection criterion (step 2 in Algorithm 1) for our reference head model *6C_Cal* and use the same spikes for the other head models.

**Figure 5:**
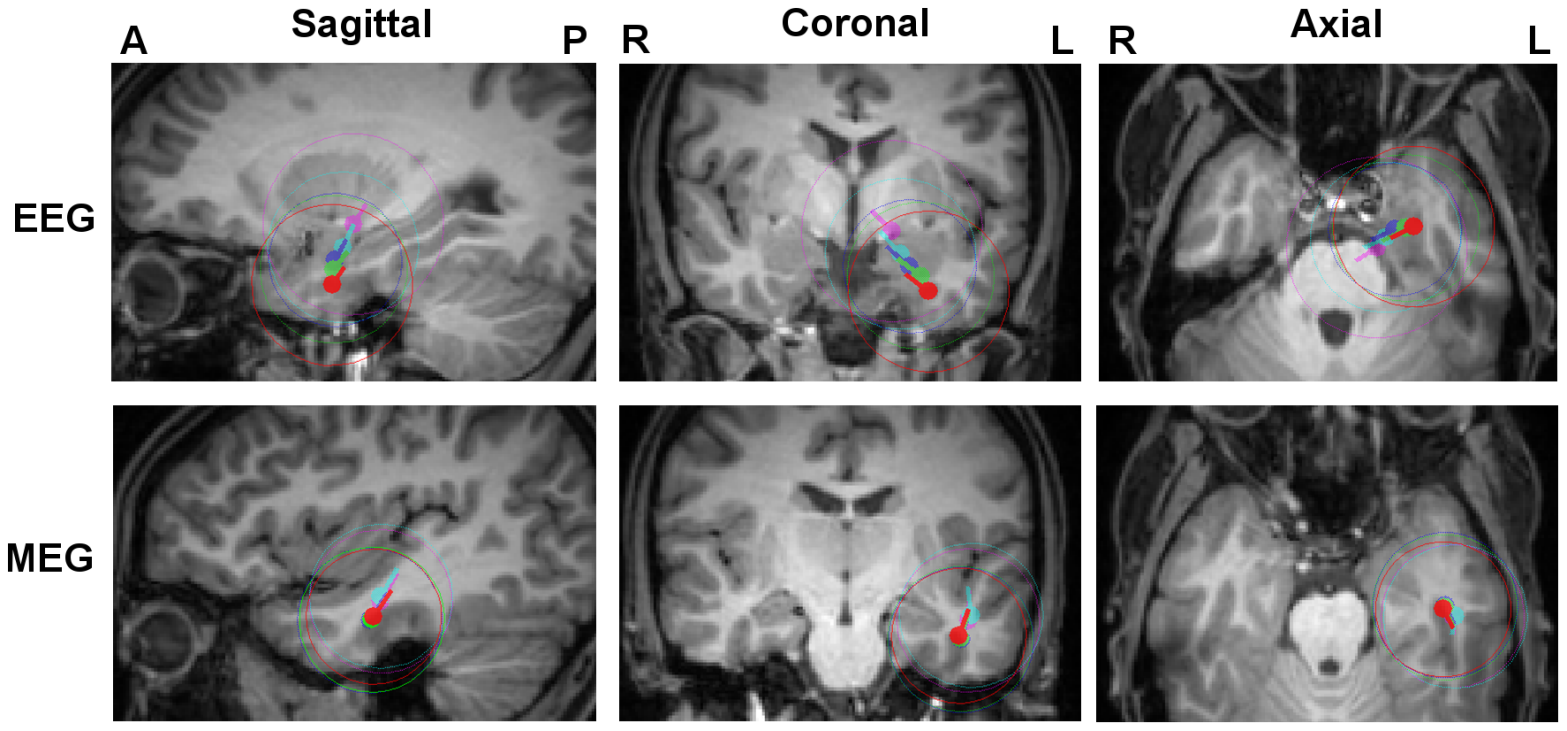
Influence of skull conductivity on EEG and MEG localizations. FT9 centroids and spread spheres plotted on T1w-MRI for head models *6C_Cal* (red), *6C_41* (green), *6C_70* (blue), *6C_132* (cyan) and *6C_330* (magenta). The centroid locations of *6C_Cal* were used for the selection of MRI slices and all results were projected on these slices.

In Figure 5, the resulting centroids and spread spheres for the FT9 cluster are plotted on the T1w-MRI. Results for the F9 cluster are very similar (see Table 2) and therefore not shown in Figure 5. We used the *6C_Cal* centroid location for the selection of sagittal, coronal and axial MRI slices and projected the color-coded results for the different head models on the chosen slices.

**Table 2:** Sensitivity of EEG and MEG spike source reconstruction with regard to skull conductivity: Differences in centroid location, orientation and strength for FT9 and F9 spike clusters for different head models from Table 1 when compared to the results achieved for the reference head model *6C_Cal*.

Spike type	Head Model	EEG			MEG		
		Location Diff. (mm)	Orientation Diff. (degree)	Strength Diff. (%)	Location Diff. (mm)	Orientation Diff. (degree)	Strength Diff. (%)
FT9	6C_41	5.7	2	−33	1.2	2	1
	6C_70	10.1	4	−45	2.0	4	0
	6C_132	15.5	11	−57	8.0	23	−10
	6C_330	23.8	8	−66	6.2	15	−13
F9	6C_41	6.0	3	−20	1.2	5	−4
	6C_70	9.9	4	−36	1.6	12	−8
	6C_132	14.5	6	−49	5.3	19	−1
	6C_330	21.1	5	−61	5.4	15	−3

Table 2 complements Figure 5 in quantifying the differences in FT9 and F9 spike cluster centroid results in terms of location, orientation and strength. In Table 2, results in head model *6C_Cal* are used as the reference and are compared to the results of the other six compartment head models.

For the EEG, as Figure 5 and Table 2 show, we observe the clear and systematic trend that, with increasing conductivity, the spike cluster centroids are localized deeper (here more mesial and superior) in the brain, while their strengths decrease. For the model with the highest conductivity *6C_330*, the centroid locations get deeper by 23.8 mm and 21.1 mm, and the strengths decrease by 66 and 61% for the FT9 and F9 spike clusters, respectively. The changes in orientations are moderate. The mean GOF (higher than 93%) is similar for all these head models.

For the MEG, while the centroid location change for FT9 and F9 spike clusters is, compared to the EEG, very moderate, MEG results still show changes in centroid moment (maximal changes in orientation and magnitude of 23 degrees and 13%, respectively) (Figure 5 and Table 2). The MEG results do not point to any systematic sensitivity of MEG localization to skull conductivity. Even if with 8 mm maximal location change, model *6C_132* points towards a slightly more superior and posterior centroid location, no trend can be observed since the change reduces to 6.2 mm for the head model with highest conductivity (*6C_330*). Again, no indicative changes are observed in terms of mean GOF (higher than 94%) for varying conductivities.

In both EEG and MEG no clear trend in spread sphere diameters can be reported.

The Euclidian distances between EEG and MEG centroids, as well as the ratio of intersection of spread sphere volumes to their union are given in Table 3 for the six compartment head models with varying skull conductivities. For both spike types, it is clearly visible that the lower the skull conductivity, the smaller the Euclidean distance between EEG and MEG centroids (from 28.3 to 16.6 mm for FT9 and from 29.4 to 24.2 mm for F9) and the larger the ratio of intersecting spread sphere volume (from 24 to 44% for FT9 and from 13 to 30% for F9). It can thus be observed that the calibrated head model *6C_Cal* not only brings SEP and SEF data together as presented in section 2.1, but also reduces the gap (especially in depth) between the EEG and the MEG spike cluster source reconstructions. However, it is also important to note that even after calibration, the EEG centroid is still considerably more anterior than the MEG centroid.

**Table 3:** Euclidean distance between the EEG and MEG centroids (in mm) and, in parenthesis, the ratio of intersecting spread sphere volumes of EEG and MEG to their union (in percent) for FT9 and F9 spike clusters and for the different head models.

Spike Type	Head Models				
	6C_Cal	6C_41	6C_70	6C_132	6C_330
FT9	16.6 (44)	17.1 (40)	20.1 (28)	26.1 (19)	28.3 (24)
F9	24.2 (30)	24.2 (31)	26.3 (25)	29.9 (16)	29.4 (13)

**Figure 6:**
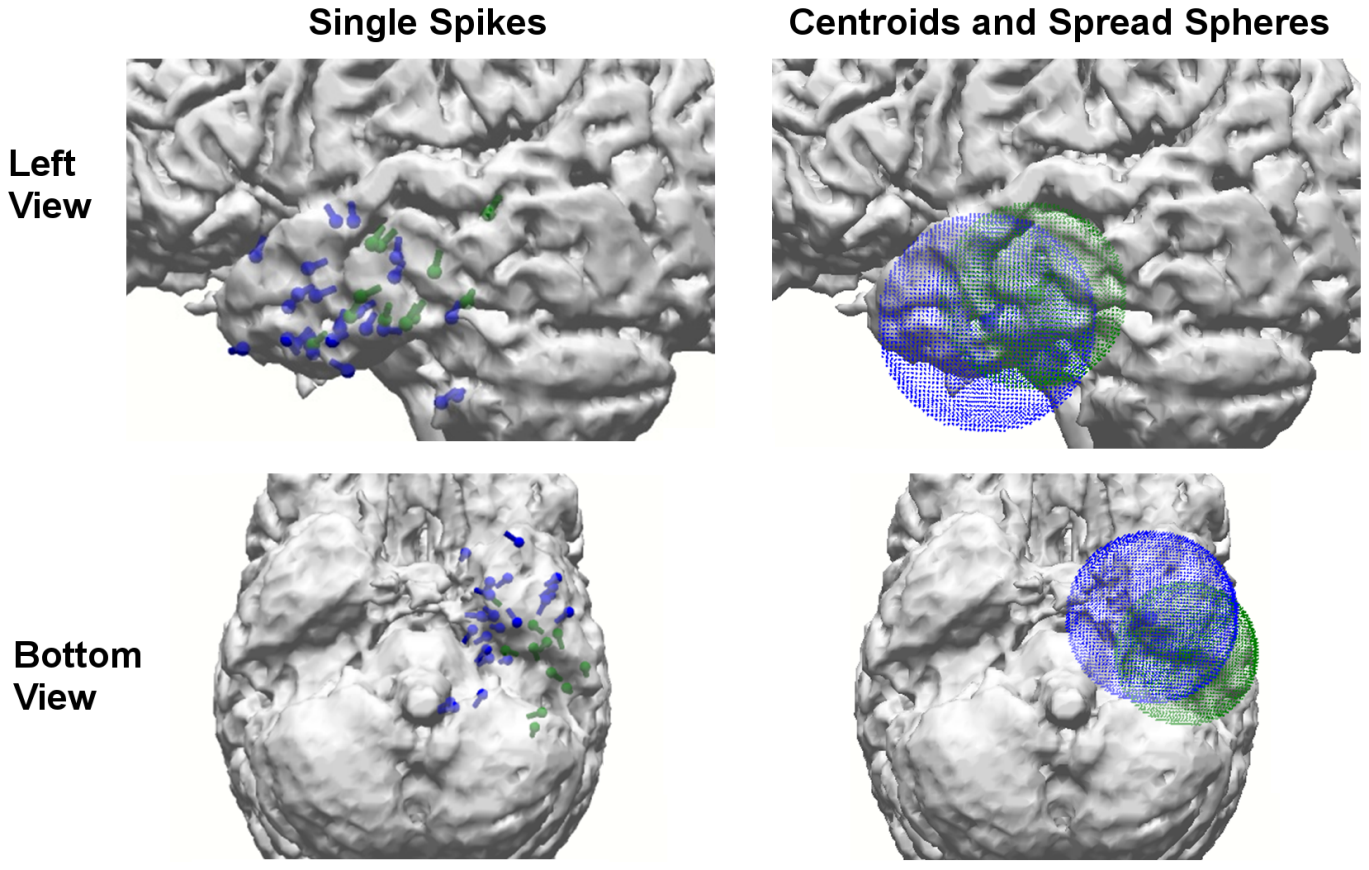
Single spike localizations and corresponding centroid and spread sphere. FT9 spike SDDS reconstructions for EEG (blue) and MEG (green) using the calibrated head model *6C_Cal* at time-point −13 ms: SDDS dipole reconstruction results of all single spikes that passed step 2 of Algorithm 1 (left) and corresponding cluster centroids and spread spheres (right).

In Figure 6, the SDDS dipole reconstructions of single spikes (that passed the GOF criterion, i.e., step 2 in Algorithm 1) (left column), as well as, the corresponding centroid and spread spheres (right column) are presented. It is clearly visible that on the one hand the EEG and MEG centroids fall inside the intersecting part of their spread spheres for the calibrated head model *6C_Cal* (optimized volume conduction can thus reduce the distance between the modalities), but on the other hand, due to the different sensitivity profiles, a remaining distance between EEG and MEG reconstructions in especially anterior-posterior direction persists.

#### 2.2.3 Effects of six versus three compartment head modeling on EEG and MEG spike source reconstruction

In this section the EEG and MEG source reconstructions using our reference individually calibrated six compartment head model *6C_Cal* are compared to the reconstructions using three compartment (3C) isotropic head models. Two 3C models, presented in Table 1, will be considered for this comparison, namely the current standard head model in source analysis, model *3C_100*, as well as the calibrated model *3C_Cal* as determined in section 2.1.

**Figure 7:**
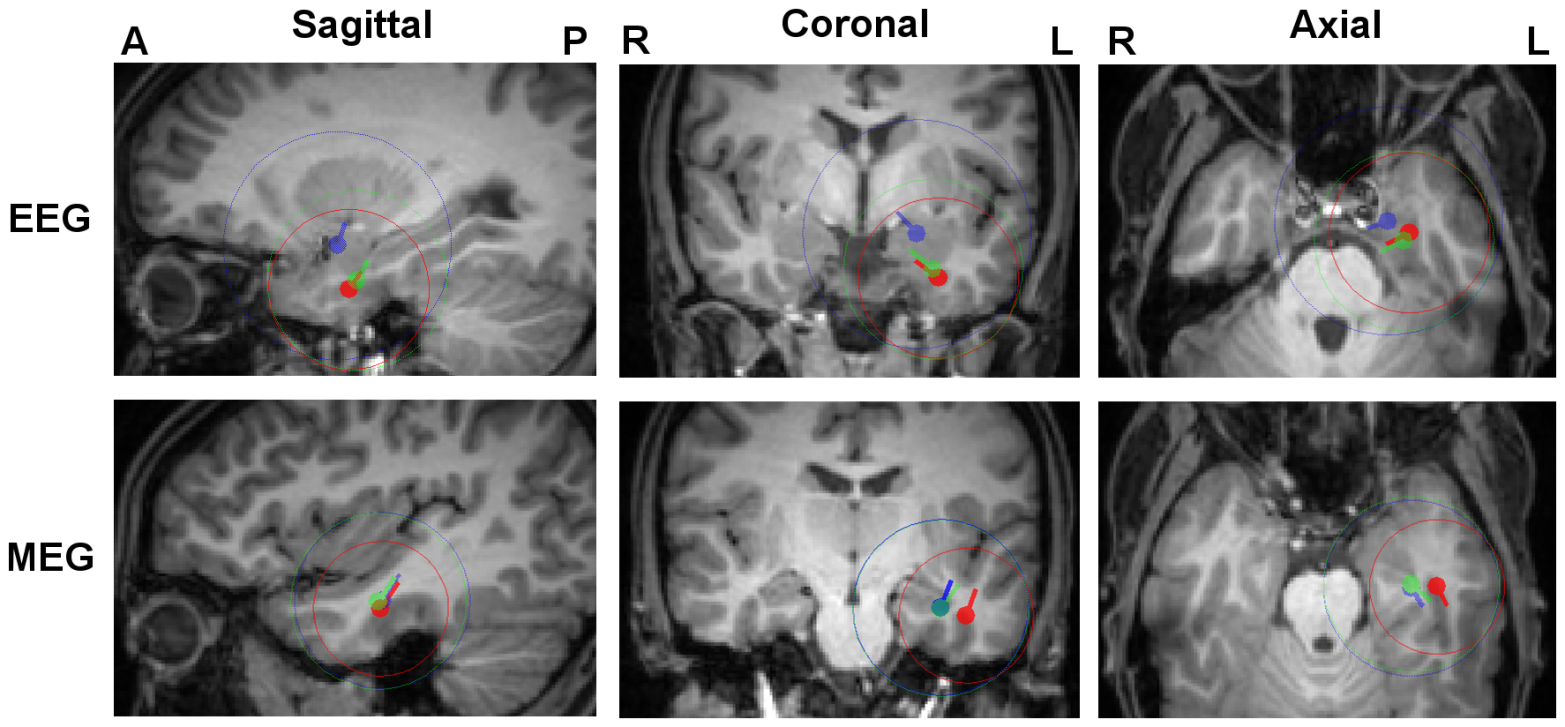
Comparison of 3 and 6 compartment head models. FT9 centroids and spread spheres plotted on T1w-MRI for head models *6C_Cal* (red), *3C_Cal* (green) and *3C_100* (blue). The centroid locations of *6C_Cal* were used for the selection of MRI slices and all results were projected on these slices.

Figure 7 shows the resulting centroids and spread spheres for the FT9 cluster plotted on the T1w-MRI. Results for the F9 cluster are very similar (see Table 4) and are therefore not shown in Figure 7. Again the *6C_Cal* centroid location was used for the selection of sagittal, coronal and axial MRI slices and the color-coded results for the different head models were projected on the chosen slices.

**Table 4:** Sensitivity of EEG and MEG spike source reconstruction with regard to three compartment (3C) or six compartment (6C) head modeling: Differences in centroid location, orientation and strength for FT9 and F9 spike clusters for the two 3C head models from Table 1 when compared to the results achieved with the reference head model 6C_Cal.

Spike type	Head Model	EEG			MEG		
		Location Diff. (mm)	Orientation Diff. (degree)	Strength Diff. (%)	Location Diff. (mm)	Orientation Diff. (degree)	Strength Diff. (%)
FT9	3C_100	16.2	13.1	−79	8.7	2.2	43
	3C_Cal	4.4	3.8	−36	8.6	15.0	67
F9	3C_100	14.6	17.8	−77	8.6	4.1	40
	3C_Cal	3.2	12.5	−28	9.1	7.0	72

Table 4 complements Figure 7 in quantifying the differences in centroid results in terms of location, orientation and strength. In Table 4, results in head model *6C_Cal* are used as the reference and compared to the results of the 3C head models.

For the EEG, for FT9 and F9 spikes the differences in centroid locations between *3C_100* and *6C_Cal* amount to 16.2 and 14.6 mm, respectively. Additionally, considerable differences in centroid orientations, much reduced centroid strengths, and strongly increased spread spheres can be reported for head model *3C_100*. Skull conductivity calibration (head model *3C_Cal*) is found to reduce these differences significantly, for centroid locations to 4.4 and 3.2 mm and orientations to 3.8 and 12.5 degrees for FT9 and F9 spike clusters, respectively. Even if the differences in centroid strengths are also reduced, with 36% and 28% magnitude reduction, differences remain at a significant level.

The situation is different for the MEG, where skull conductivity calibration has hardly any effect on the localization of the sources. Figure 7 and Table 4 show that centroids and spread spheres are nearly identical for models 3C_100 and 3C_Cal, while with about 9 mm and more than 40%, differences in location and strength are considerable for both FT9 and F9 spike clusters in comparison to 6C_Cal. Please also note for the MEG the higher strength and orientation differences for 3C_Cal in comparison to 3C_100. This only shows the weakness of MEG to accurately reconstruct radial source orientation and strength components in the presence of noise. Additionally, we can report significantly larger spread sphere diameters in 3C when compared to 6C models.

#### 2.2.4 Comparison of combined EEG/MEG to single modality EEG or MEG source reconstruction

In previous sections we gained deep insight into the factors that influence EEG and MEG source analysis with a special focus on volume conduction effects due to geometry and/or conductivity modeling changes as well as effects which were mainly due to limited SNR in measurements. We will now make use of this knowledge when studying combined EEG/MEG source analysis in comparison to single modality EEG or MEG reconstructions of the epileptic spike activity. For this comparison, we use our most advanced head model *6C_Cal* from Table 1.

**Figure 8:**
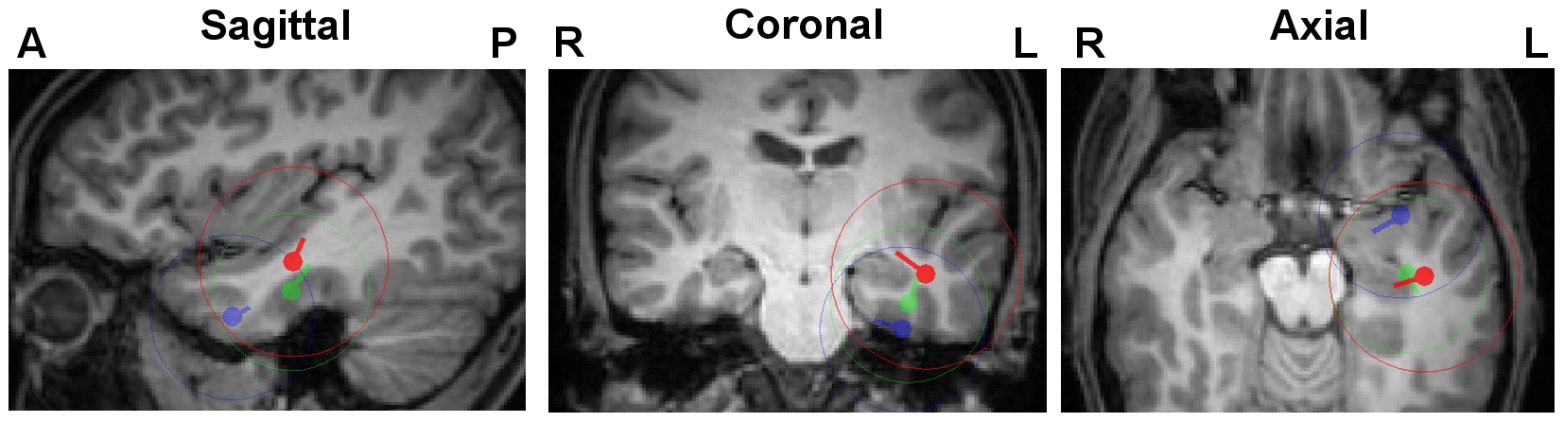
Differences of EEG, MEG and combined EEG/MEG localizations. FT9 centroids and spread spheres plotted on T1w-MRI for combined EEG/MEG (red), MEG (green) and EEG (blue) using head model *6C_Cal*. The centroid location of the combined reconstruction was used for the selection of MRI slices and all results were projected on these slices.

Figure 8 and Table 5 show the results of Algorithm 1 for FT9 and F9 spike cluster centroid and spread sphere computations for combined EEG/MEG and for the single modalities EEG and MEG. In Table 5, the combined EEG/MEG results serve as the reference and differences in centroid locations, orientations and strengths are presented for each of the single modalities, EEG and MEG.

**Table 5:** Comparing EEG and MEG spike cluster centroid results to the results of combined EEG/MEG using the reference head model 6C_Cal: Differences in centroid location, orientation and strength for FT9 and F9 spike clusters.

Spike type	Modality	Difference from Combined EEG/MEG		
		Location Diff. (mm)	Orientation Diff. (degree)	Strength Diff. (%)
FT9	EEG	24.2	26	−7
	MEG	9.8	70	−74
F9	EEG	30.9	21	63
	MEG	9.2	79	−78

Figure 8 and Table 5 show that the combined EEG/MEG centroids are localized about a factor of 2.5 (FT9) and even about 3.4 (F9) closer to the MEG than to the EEG centroid locations. However, with 9.8 mm for FT9 and 9.2 mm for F9, there is still a considerable distance between the combined EEG/MEG and the MEG centroid localizations. The localization was thus not just totally dominated by the MEG, but was rather a complicated interplay of a main MEG and still a considerable EEG part, pointing to a considerable radial source component as also clearly visible in Figure 8. This brings us to the evaluation of combined EEG/MEG source orientation and strength results, which are influenced more by the EEG part, as Figure 8 and Table 5 clearly show, while with orientation differences of 70 degrees and more, it gets clear that the MEG is mainly missing the radial source component.

As Figure 8 shows, the spread sphere diameters of combined EEG/MEG (29 mm) were slightly larger than those of EEG (25 mm) and MEG (24 mm).

As a final result, Table 6 shows the differences in centroid reconstructions in combined EEG/MEG scenarios, using the six compartment models of Table 1 instead of the reference head model *6C_Cal*. This table shows a clear trend of increasing source location differences with increasing skull conductivity. A more detailed analysis showed that higher skull conductivity led to deeper source localizations, i.e., similar to the EEG centroid results in Figure 5, quasi-radially into the deeper brain regions. However, with maximal differences of 7.8 mm (FT9) and 13.9 mm (F9) for the head model with highest skull conductivity (*6C_330*), the differences are considerably lower than for the EEG (23.8 mm for FT9 and 21.1 mm for F9, see Table 2). Table 6 shows decreasing source strength with increasing skull conductivity, but with 62% (FT9) and 52% (F9) for model *6C_330*, the reductions are smaller than for the EEG (66% for FT9 and 61% for F9, see Table 2). Interestingly, Table 6 now additionally shows a clear and systematic trend of increasing orientation differences with maximums as 17 degrees (FT9) and 13 degrees (F9) for model *6C_330*, while such a trend could not be observed for the EEG in Table 2. A more detailed analysis (using the singular value decomposition of the MEG lead field matrix to determine the quasi-radial orientation component) revealed a decreasing quasi-radial and a constant quasi-tangential centroid component with increasing skull conductivity. The GOF for model *6C_Cal* for combined EEG/MEG is 95% (FT9) and 93% (F9). As Table 6 shows, for FT9 spikes, the GOF stays mainly on this high level for all 6C head models, while for the F9 spike cluster, a trend towards decreasing GOF with increasing skull conductivity can be noted with a 6% reduction, i.e., only 87% GOF, for model *6C_330*.

**Table 6:** Sensitivity of combined EEG/MEG spike source reconstruction with regard to skull conductivity: Differences in centroid location, orientation, strength and GOF for FT9 and F9 spike clusters for different head models from Table 1 when compared to the results achieved for the reference head model 6C_Cal.

Spike type	Head Model	Combined EEG/MEG			
		Location Diff. (mm)	Orientation Diff. (degree)	Strength Diff. (%)	GOF Diff. (%)
FT9	6C_41	1.8	3	−26	0
	6C_70	1.7	8	−55	0
	6C_132	3.2	13	−56	0
	6C_330	7.8	17	−62	−1
F9	6C_41	3.3	1	−24	0
	6C_70	6.1	9	−40	−1
	6C_132	11.6	11	−42	−4
	6C_330	13.9	13	−52	−6

## Discussion

In this study we presented a new analysis pipeline for combined EEG/MEG as well as single modality EEG or MEG source reconstruction based on a calibrated realistic head model generated from T1w-, T2w- and DTI data. Inspired by [29], [30], [72], [73], we developed and applied an algorithm (Algorithm 2 in section 1.11) for skull conductivity calibration using simultaneously acquired SEP/SEF data. The measurement time, which was divided as one block for EEG/MEG (7 minutes for SEP/SEF, plus 40 minutes for spontaneous epileptic activity) and one for MRI (27 minutes), was easily manageable for the patient. As input, this procedure needs an accurately segmented model of the head, and in particular, a geometrically correct version of the skull. Whereas computer tomography provides better definition of hard tissues such as bones due to high radiation exposure, its use on humans is not justified with the only purpose of an improved skull modeling for EEG and MEG source analysis [13], [35], [38]. In this study, we used a combination of T1w-MRI, which suits to the identification of soft tissues (scalp, brain), and T2w-MRI, enabling the segmentation of the inner skull surface and the distinction between skull compacta and spongiosa. The methodology was then applied in a case study to source analysis of interictal epileptic activity of a patient suffering from medically-intractable epilepsy, but could as well be used for any other simultaneous EEG/MEG study in the neuroscientific field (the short additional measurement time, which was easily manageable even for our patient, should not form an obstacle in a group study with healthy subjects). In our investigations, we used a variety of head models which differed in terms of skull conductivity or in the number of distinguished tissue types (Table 1). Our most advanced head model, the six compartment (6C) calibrated model *6C_Cal*, consists of the tissues skin, skull compacta, skull spongiosa, CSF, gray and white matter, uses the individually-optimized skull conductivity parameters from the calibration procedure, and accounts for the anisotropy of the brain tissues. Our method considers the different sensitivity profiles of the EEG and MEG to properties of the volume conductor and source components (see also [27], [29]). Therefore, before investigating combined EEG/MEG scenarios, we studied important parameters that influence EEG and/or MEG source reconstruction.

Our first investigation focused on a comparison of EEG and MEG with regard to a parameter to which they have the most distinct sensitivity and which, as shown in Table 1, has a considerable interindividual variability: the skull conductivity. For the same underlying source, due to different sensitivity profiles in volume conduction, the differences between EEG and MEG source reconstructions could increase in case of an erroneously modeled skull compartment. Therefore, we propose a multimodal MRI procedure for skull geometry modeling and Algorithm 2 based on SEP/SEF data to individually estimate skull conductivity. We then applied the new methodology to the reconstruction of the SEP and SEF N20 component (section 2.1) and to the spontaneous interictal epileptic activity (section 2.2.2). We found that for the MEG, skull conductivity changes had no effect in terms of N20 localization, but had non-negligible effects on source orientation and strength. This can be explained with the well-known instability of MEG in reconstructing quasi-radial source magnitude. In contrast, EEG results differed significantly in terms of N20 location, orientation and strength: the higher the skull conductivity, the deeper the localization and the smaller the source magnitude. Besides the differences (6C versus 3C) discussed further below, these results are therefore mainly in agreement with former 3C head modeling approaches [7], [29], [30]. For the epileptic activity, we compared EEG and MEG, and investigated the effects of varying skull conductivities in section 2.2.2. These results further confirm our findings. For the EEG, a clear trend of deeper source localizations and reduced source amplitudes can be reported with increasing skull conductivity. Table 2 showed that location differences of more than 21 mm can result in case of erroneously chosen skull conductivity. MEG source reconstructions of the epileptic activity did not show a trend similar to EEG and the reconstruction differences with changing conductivity were significantly smaller. A closer look at the largest MEG centroid localization change in Table 2 (model *6C_132*) confirmed that this difference is not a consequence of a systematic sensitivity of MEG to skull conductivity changes, but mainly due to the interplay of the high noise in spike data with the chosen procedure of centroid calculation, namely preselecting single spikes with regard to their SNR and GOF, performing single spike and single dipole deviation scans (SDDSs), and averaging the global peak of the resulting GOF function for computing spike cluster centroids. As explained above, MEG orientation and strength components should also be interpreted with caution because of the poor sensitivity of MEG to radial source components.

Let us now focus on the distance between EEG and MEG localizations: Table 3 demonstrates that the skull conductivity calibrated model *6C_Cal* reduces the distance (especially the difference in depth) between EEG and MEG localizations and maximizes the ratio of the intersecting spread spheres. However, localization differences might still resist like in our case, and these discrepancies can be explained by the different sensitivity profiles of EEG and MEG, where MEG mainly sees the more tangential parts of an extended cortical patch (the more posterior localization in our results) and EEG more the radial parts (the more anterior polar localization in our results), as also discussed by [74] and [75].

Another goal of our study was making a comparison between 6C and 3C head modeling. Our model *3C_100* can be considered as the current standard in source analysis [6], [35]. For the reconstruction of the N20 component in the SEP and SEF scenarios in section 2.1, we found significant differences between *3C_100* and *6C_Cal* reconstructions for both EEG and MEG. While skull conductivity calibration (model *3C_Cal*) brought no significant change for the MEG (i.e., the MEG differences between *6C_Cal* and *3C_Cal* remained at a significant level), it enabled us to reduce depth localization differences for EEG considerably, while differences in source orientation and strength persisted. In the case of epileptic activity a similar behavior has been observed (section 2.2.3). For MEG, significant differences can be reported between *6C_Cal* and *3C_100*, which could again not be reduced by means of skull conductivity calibration. Even if, for the EEG, up to 16 mm differences in centroid locations between *6C_Cal* and *3C_100* could be reduced to less than 5 mm between *6C_Cal* and *3C_Cal*, considerable differences in centroid orientations and strengths persisted. We can therefore summarize that, for EEG localization, skull conductivity is the dominating parameter, while the highly conducting CSF and brain anisotropy contribute significantly to EEG and MEG source orientation and strength components (see also [33], [40], [42], [50], [52]). If the sources have a considerable radial orientation component like in case of our spike data, CSF and brain conductivities can additionally influence MEG localization (about 9 mm in Table 4), but the more quasi-tangential the source is, the less MEG is influenced by these parameters (less than 5 mm for the N20 SEF reconstruction). While the modeling of skull inhomogeneity by means of a distinction between skull compacta and spongiosa might be important for EEG in other situations [35], it was not a crucial factor here (see Figure 3 and section 2.2.3), because the major spongiosa areas were far from the central and temporal source space areas for this patient (see coronal slice in Figure 1).

The effects of using different head models were found to be significantly higher for the epileptic activity in the temporal area in comparison to the somatosensory evoked responses. In the light of the existing literature (see, e.g. [12], [29], [33], [35], [76]), this is not too astonishing. For example, in [76], the comparison of a spherical with a 3C realistically-shaped head model clearly showed larger MEG volume conduction effects for fronto-temporal and deep sources. Huiskamp et al. [12] showed that EEG sources arising from temporal regions are especially susceptible to geometrically inaccurate skull models. Possible explanations are: a) the skull in the temporal area has a higher concavity than in the area of the central sulcus, leading to larger volume conduction effects; b) the underlying source of the SEP/SEF N20 component is mainly a single superficial dipole with quasi-tangential orientation where especially MEG is very sensitive to and therefore less prone to errors due to simplifications in volume conduction (see Figure 3). In contrast, both FT9 and F9 temporal spike sources were deeper and had a considerable radial orientation component, rendering especially the MEG more susceptible to volume conduction effects; c) the EEG and MEG sensor coverage is much better above the central sulcus, where both poles are clearly visible in the SEP/SEF data. For the spikes in temporal lobe, some of the activity which was supposed to appear at inferior regions could not be measured due to the limited coverage of basal brain regions with the used EEG cap.

Our results in sections 2.1 and 2.2.4 clearly show that the combined EEG/MEG centroid results profit from the MEG which contains important localizational information for the tangential source components, an information which is even not depending much on the accuracy of skull (and skin) modeling. On the other hand, the combined EEG/MEG centroid results profit from the EEG, which could add the information that was mainly missing in the MEG, namely the localizational information about the radial source components, and the full information on source orientation and strength components (see also [7], [27]–[31], [33]). However, the latter statement has the constraint of an underlying accurate and individually-calibrated head volume conductor model, since with more than 21 mm localization differences (see Table 2) we found EEG localizations to highly depend on skull conductivity parameters in accordance with the literature [35], [77].

Source localization techniques have error margins that are proportional to the inverse of the SNR. Since single spike activity has a significantly lower SNR than averaged somatosensory responses, its localization is less reliable and therefore not always sufficient for precise localization of the epileptic tissue. It has, however, been reported that also the orientation of the dipole possesses localizational information regarding the epileptic tissue [13], [15]. In [13] the importance of dipole orientation for temporal spikes was stressed, where the authors showed different seizure freedom ratios for patients with horizontally and vertically oriented dipoles. In [15] all central and interhemispheric, and 73% of the temporal spike dipoles (positive part) were observed to be oriented towards the epileptogenic side. The MEG source orientations in section 2.2.4 were almost orthogonal to the combined EEG/MEG orientations, because MEG could hardly measure the quasi-radial orientation components of the underlying sources. Combined EEG/MEG thus contains information which is missing in single modality EEG or MEG and this information can be exploited to achieve improved source reconstructions not only with regard to localization, but also with regard to source orientation [13], [15]. However, as we have shown in our comparisons, especially source orientation and strength components are susceptible to simplifications or modeling errors with regard to the CSF and brain compartments and in many situations, the distinction between skull spongiosa and compacta might be of high importance [35], too. These arguments underline the need to further validate and evaluate the accuracy of anisotropic 6C volume conductor modeling in future investigations.

The results of combined EEG/MEG in the presence of erroneously chosen skull conductivity (Table 6) can be interpreted in the following way: The MEG part of the combined EEG/MEG dataset stabilized especially the depth localization. Localizations quasi-radially into the depth of the brain could be much reduced (e.g., for the FT9 spike cluster centroid from 23.8 mm for EEG in Table 2 down to 7.8 mm for combined EEG/MEG in Table 6). In order to simultaneously achieve a high GOF to both datasets, the strength of the radial centroid component was reduced for higher skull conductivities (by means of a significant reduction of overall centroid strength and an orientation change towards more quasi-tangential orientation). In this way, high GOF to the EEG data could still be achieved, while keeping the magnitude of the tangential source component mainly unchanged in order not to change GOF to the MEG data (Table 6). Because of the distinct quasi-tangential orientation component of the FT9 spikes, this procedure worked out nearly without any loss in GOF to the combined EEG/MEG data, even in case of highly erroneous skull conductivity. However, GOF reduced by 6% for the F9 spikes because of their more distinct quasi-radial orientation component. The comparison of the results presented in Table 6 with those in Table 2 thus represent an advantage of combined EEG/MEG versus single modality EEG or MEG in practical situations: In case of a moderate error in skull conductivity modeling, combined EEG/MEG source analysis can still profit from the strength of the MEG to accurately localize the tangential source component while the EEG can still contribute much to better localize radial source components, and determine source orientation and strength (see also Figure 8 and Table 5). On the other side, significant errors in skull modeling will be reflected by a complicated interplay of errors in location (especially in depth), orientation and strength of EEG sources, and in the worst case a significantly reduced GOF to the combined EEG/MEG datasets. We therefore recommend calibrating skull conductivity using additionally acquired SEP/SEF data.

As described in detail in section 1.6, in a first step, three epileptologists used a subset of electrodes to mark the epileptic activity based on the current clinical agreement. In a second step, and using the complete set of sensors, the spikes were then clustered according to the electrode where the maximum negativity in referential montage (common average) occurred. In this way, we found 2 different spike types, FT9 and F9, which mainly differed with regard to their orientation components. This shows that it might not be sufficient to use the subset of sensors in step one also for step two, the clustering. For example, if our clustering montage did not have an FT9 but just an F9 electrode, FT9 spikes would have been clustered as F9 because the evaluator would have seen the maximum negativity at this electrode. Such issues in clustering process might cause errors in centroid as well as in spread sphere computations. When using spike averaging, it would lead to smeared peaks and SNR reductions. For the purpose of this study, our clustering procedure led to satisfying results. However, in future examinations, we are intending to also evaluate other concepts such as source montages [78].

Two approaches are mainly used for determining the irritative zone. The first approach (and the one we used in this study) is to reconstruct each single spike separately and determine the irritative zone according to the clusters that those spikes produce. The second approach is to average the spikes that belong to the same class (i.e., they have a sufficiently similar EEG/MEG topography) in sensor space and then perform source reconstruction. The advantage of the latter approach is that it allows an improved SNR if enough spikes of the same class can be found and averaged [8], [16]. The sources obtained in this way from the averaged spikes are a collection of the underlying focal sources and represent a considerable portion of the irritative zone. However, in [64], [65] it was shown that the activated cortical areas during sharp waves are focal and their spatial positions change in a dynamic manner. Even though not all sharp waves can be detectable with extracranial recordings [79], the appeal of the first approach is that the spreading of the localizations might give an estimate on the focality of the irritative zone [66], [67], as also investigated using spread spheres in this study. In order to avoid effects that are just due to insufficient SNR, we have considered here only the spikes with minimal SNR of 3 and GOF of 91% (Algorithm 1). In this way, and despite the still low SNR in our single spike data and the resulting lower reliability of source reconstruction results, we could clearly work out EEG, MEG as well as combined EEG/MEG volume conduction effects on the reconstruction of the spike clusters. These volume conduction effects thus dominate over higher noise and need to be taken into account even in single spike source analysis, while they should appear in an even purer form and accordingly be taken into account when working with averaged spike data. Motivated by the results of [8], in future studies, we are thus intending to investigate volume conduction effects in EEG, MEG and combined EEG/MEG studies using single spike versus averaged spike reconstructions.

A further important choice when reconstructing epileptic spike activity is the selection of the time-point or time-interval for the localization of the spikes. The peak of the spike indicates the highest degree of neuronal synchronicity and thus better SNRs, but on the other hand this location might already have been subject to propagation. Therefore, we selected here the middle of the rising flank from the averaged spikes as a time-point for later single spike reconstructions because it was shown to be favorable when compared to the reconstruction at the peak of the spike [68]. We assume that due to higher SNR at the peak of the spike, the presented volume conduction effects could probably be presented in an even clearer form (e.g., the presented effects on MEG in Table 2 and 4 were found to be at least partly due to the high noise level and not only due to MEG volume conduction effects). Since reconstructions will be dominated more and more by noise when approaching the area of low SNR at the beginning of the spike, at such early time-points, a combination of the here presented methodology with spike averaging strategies seems to be mandatory.

We have shown that by means of using a calibrated six compartment head model, we could already significantly reduce the distance in localization, orientation and strength between EEG and MEG centroids as well as increasing the intersection of their spread spheres (see Table 3 and section 2.2.4). Reasons for the remaining distance between EEG and MEG reconstructions are the following: a) None of the single modality EEG or MEG contains the full information about the sources, MEG mainly misses the quasi-radial source components and for low SNRs EEG is rather weak in reconstructing the quasi-tangential ones. A remaining difference thus should be expected even with the best head modeling. This problem can be reduced when fusing both modalities in combined EEG/MEG source analysis, as described in section 2.2.4. b) Our Polhemus-procedure for EEG sensor registration, our fiducials based procedure for morphing EEG and MEG onto the MRI and patient movements in EEG/MEG and in MRI cause artifacts, which are reflected in persisting differences of EEG and MEG reconstructions. c) Even if we already invested much in creating a patient-specific realistic volume conductor model, our model still contains simplifications and modeling errors as explained in the following paragraph.

In this study we tried to keep the manual intervention to the segmentation results to minimum and intended to offer a modeling pipeline that uses the outputs of freely available programs. For this reason, we did not include skull holes to our model. Skull holes were shown to have significant effects in both EEG [38] and to a lower extent in MEG [80]. However, these studies also showed that for small holes the errors tend to be limited to the close vicinity of the hole. In our study, we assumed that the SEP/SEF and the spike sources are sufficiently far away from skull holes, so that effects (e.g., from the foramen magnum) should be negligible. However, in case of craniotomy, where the hole is near the brain region of interest, it becomes far more important and should be modeled for reliable source reconstruction [81]. Another simplification used in our study is a slight overmodeling of the outer CSF space as the space between inner skull surface and brain surface (see Fig.1). We therefore did not explicitly model the meninges, because of the unknown conductivities [82], the low thicknesses of these structures and, as a consequence, their difficult segmentation. Even if it has been shown that dura mater affects the EEG [83], it has an average thickness of only 0.36 mm [84] and contains many blood vessels. The subarachnoid space is filled with CSF, has an average thickness of 3.1 mm [84] and its conductivity is well-known and intra- and inter-individually stable [39]. The overmodeled CSF might lead to slightly bigger differences between three and six compartment models and slightly lower skull conductivities in the calibration procedure. Since this procedure is not intended to find the exact conductivity of the skull but rather to calibrate it for the used head model, we do not assume significant changes to the overall results.

Although our results show major effects of skull conductivity calibration as proposed here, not all institutes have access to MEG. In these cases, for estimating skull conductivity, we would suggest either using a similar procedure based on only good quality SEP data as proposed in [69] or an electrical impedance tomography (EIT) based approach as described by [85].

Our study showed that even for single interictal spikes EEG and MEG volume conduction effects dominate over noise and need to be taken into account for accurate source analysis. EEG and MEG contain complementary information and a simultaneous acquirement of both datasets is recommended to increase reliability of results not only in presurgical epilepsy diagnosis, but also in other neuroscientific application fields. The time for acquiring the additional data is easily manageable by the patient and should not form an obstacle for the proposed procedure even in clinical group studies, intended to be carried out by us in the near future. We presented a proof of concept that skull conductivity calibration in realistic six and three compartment head models is needed to accurately combine EEG and MEG source analysis.
